# The INFluence of Remote monitoring on Anxiety/depRession, quality of lifE, and Device acceptance in ICD patients: a prospective, randomized, controlled, single-center trial

**DOI:** 10.1007/s00392-020-01667-0

**Published:** 2020-05-16

**Authors:** Florian Leppert, Johannes Siebermair, Ulrich Wesemann, Eimo Martens, Stefan M. Sattler, Stefan Scholz, Stefan Veith, Wolfgang Greiner, Tienush Rassaf, Stefan Kääb, Reza Wakili

**Affiliations:** 1 School of Public Health, Bielefeld University, Bielefeld, Germany; 2 Department of Medicine I, University Hospital Munich, Ludwig Maximilians University, Munich, Germany; 3 Department of Cardiology and Vascular Medicine, West-German Heart and Vascular Center Essen, University of Essen Medical School, University Duisburg-Essen, Essen, Germany; 4 Deutsches Zentrum für Herz-Kreislauferkrankungen (DZHK), Partner Site Munich Heart Alliance, Munich, Germany; 5 Department of Psychiatry, Psychotherapy and Psychotraumatology, Bundeswehr Hospital, Berlin, Germany; 6 Medizinische Klinik und Poliklinik, Klinikum rechts der Isar, Technische Universität München, Munich, Germany; 7 Department of Cardiology, Heart Centre, Copenhagen University Hospital, Rigshospitalet, Copenhagen, Denmark

**Keywords:** Quality of life, QoL, Remote device monitoring, Telemedicine, Implantable cardioverter–defibrillator

## Abstract

**Background:**

Impact of telemedicine with remote patient monitoring (RPM) in implantable cardioverter–defibrillator (ICD) patients on clinical outcomes has been investigated in various clinical settings with divergent results. However, role of RPM on patient-reported-outcomes (PRO) is unclear. The INFRARED-ICD trial aimed to investigate the effect of RPM in addition to standard-of-care on PRO in a mixed ICD patient cohort.

**Methods and results:**

Patients were randomized to RPM (*n* = 92) or standard in-office-FU (*n* = 88) serving as control group (CTL). At baseline and on a monthly basis over 1 year, study participants completed the EQ-5D questionnaire for the primary outcome Quality of Life (QoL), the Hospital Anxiety and Depression Scale, and the Florida Patient Acceptance Survey questionnaire for secondary outcomes. Demographic characteristics (82% men, mean age 62.3 years) and PRO at baseline were not different between RPM and CTL. Primary outcome analysis showed that additional RPM was not superior to CTL with respect to QoL over 12 months [+ 1.2 vs. + 3.9 points in CTL and RPM group, respectively (*p* = 0.24)]. Pre-specified analyses could not identify subgroups with improved QoL by the use of RPM. Neither levels of anxiety (− 0.4 vs. − 0.3, *p* = 0.88), depression (+ 0.3 vs. ± 0.0, *p* = 0.38), nor device acceptance (+ 1.1 vs. + 1.6, *p* = 0.20) were influenced by additional use of RPM.

**Conclusion:**

The results of the present study show that PRO were not improved by RPM in addition to standard-of-care FU. Careful evaluation and planning of future trials in selected ICD patients are warranted before implementing RPM in routine practice.

**Graphic abstract:**

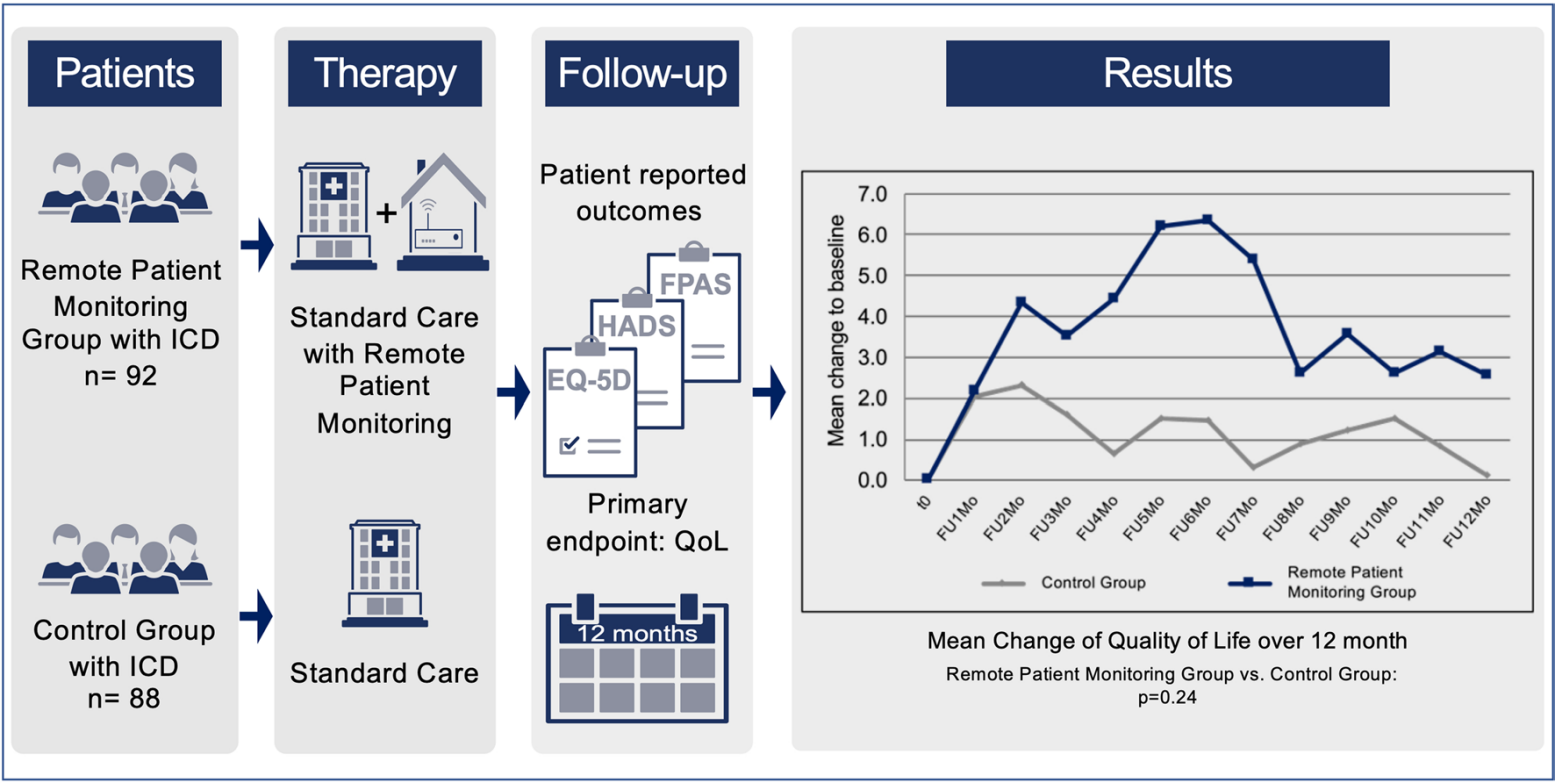

**Electronic supplementary material:**

The online version of this article (10.1007/s00392-020-01667-0) contains supplementary material, which is available to authorized users.

## Introduction

Shocks of implantable cardioverter–defibrillators (ICDs) for ventricular arrhythmias are associated with worse patient-reported outcomes including feelings of depression, anxiety, and loss of control [1]. Although therapy with ICDs is proven to be effective in the prevention of sudden cardiac death [2], its use in clinical practice is currently facing problems of limited resources in the health care system. This led to the development of new ICD follow-up (FU) strategies with active patient involvement, namely remote patient monitoring (RPM) for telemetric transmission of ICD data. However, the majority of recent large randomized-controlled trials has failed to provide consistent evidence for the superiority on hard clinical endpoints, i.e., mortality [3–5]. Additional potential effects of RPM, such as improvement of patient-reported outcomes, are not well investigated [6]. Therefore, we investigated the effect of RPM in addition to standard in-office ICD follow-up on patient-reported outcomes compared to patients receiving standard in-office ICD follow-up only. The change of Quality of Life (QoL) over 12 months (measured by EQ-5D-index) served as the primary endpoint.

## Methods

### Trial design and participants

This controlled longitudinal randomized open-label trial was conducted at the Ludwig Maximilians-University of Munich, Campus Grosshadern. Design of the trial, including questionnaire use, and an assessor-blinded analysis were performed in collaboration with the Bielefeld University, School of Public Health. The study was approved by the local ethics committee (registration no. 166-11) and registered at clinicaltrials.gov (Identifier: NCT02888028).

**Fig. 1:**
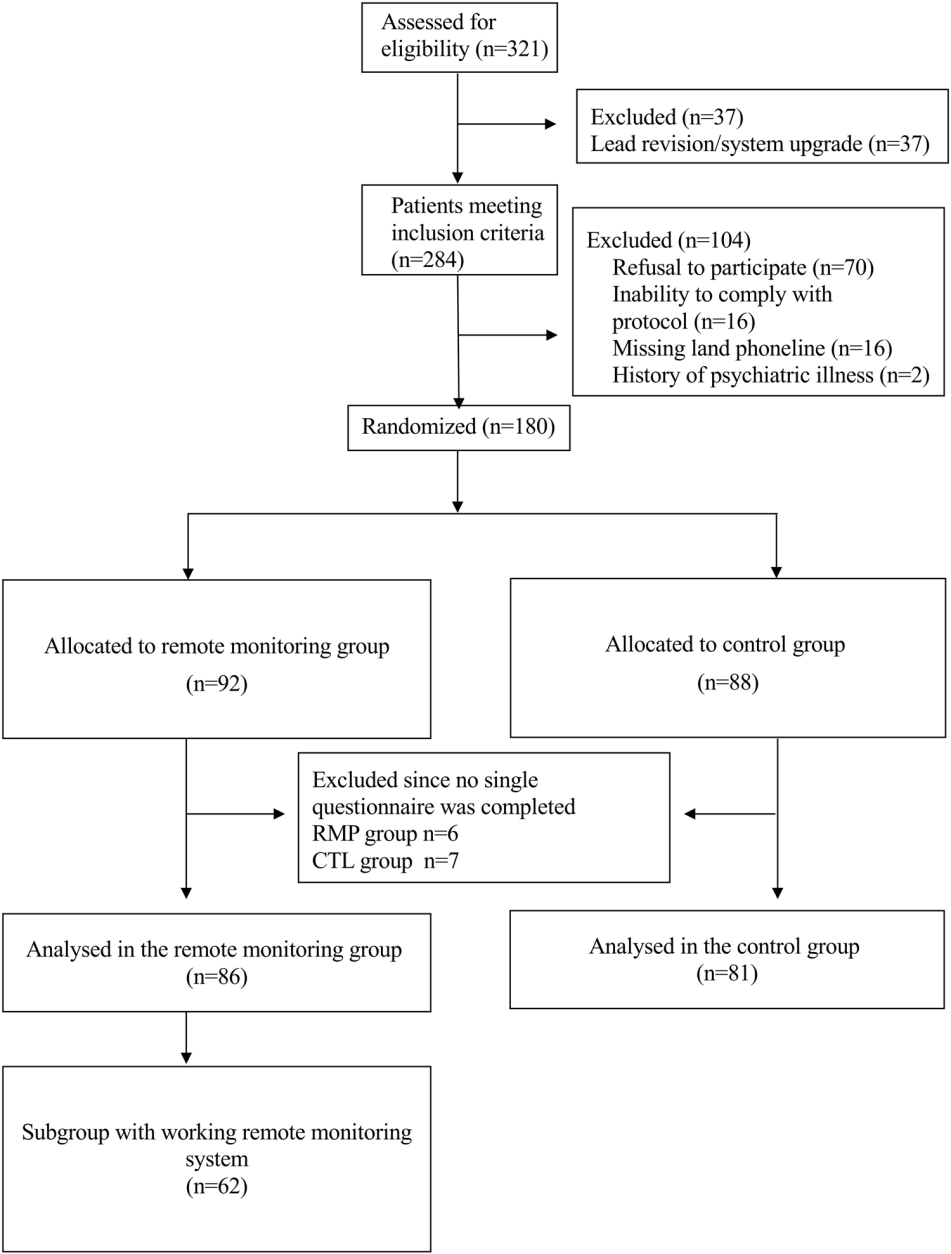
CONSORT statement flow diagram according to study protocol for both study cohorts. For the intention-to-treat analysis, *n* = 86 patients in the RPM group and *n* = 81 patients in the CTL group were analyzed with respect to patient-reported outcomes. *CTL* control, *RPM* remote patient monitoring

All patients aged 18 years and older presenting to our clinic for new implantation or replacement of an ICD due to battery depletion were eligible for inclusion (*n* = 321). The decision which system to implant was related to the discretion of the treating physician considering the different pacing and VT discrimination algorithms, sensor techniques, size, and battery longevity of the individual systems. The brands of the ICD systems implanted were as follows: Medtronic *n* = 71; Biotronik *n* = 49; St. Jude Medical *n* = 34; and Boston Scientific *n* = 26. Following assessment of exclusion criteria (refusal to participate, insufficient language skills, inability to comply with the protocol, a history of severe psychiatric illness, or missing availability of a standard land phone line) and informed consent, a total of 180 patients were included in the study and followed for 12 months (see Fig. 1).

### Intervention

Patients were randomly assigned to either routine standard in-office FU serving as control group (CTL) or the interventional arm with RPM in addition to in-office FU. Using a block randomization provided by the Bielefeld University, the University of Munich was provided with the randomization result after successful enrollment of the patient.

In both groups, standard in-office FU every 3–6 months was performed. Patients in the RPM group received an RPM system in addition to the standard in-office FU to evaluate the “pure” additional effect of RPM. Patient-reported outcomes were measured at baseline and monthly during the 12-month FU period.

### Remote patient monitoring implementation and training process

Patients were informed of the advantages and disadvantages of the different means of ICD follow-up, with a special regard on RPM surveillance (advantages: reduced number of in-office visits, option of extended follow-up periods, early detection of ICD malfunctions, and use of heart-failure monitoring tools; disadvantages: no option of telemetric ICD re-programming).

According to the different brands of implanted ICDs, four different RPM systems were used in the present study, those were the CareLink™ system (Medtronic), HomeMonitoring™ (Biotronik), the Latitude™ (Boston Scientific) and the Merlin.net™ system (St. Jude Medical). Data that were transmitted comprised battery status, sensing parameters, stimulation and high-voltage impedance, information on applied ICD therapies (shocks and/or antitachycardia pacing), and transmission of additional, device-specific diagnosis algorithms, like information on heart rate profile/variability, percentage of stimulation or atrial fibrillation burden. Except for the HomeMonitoring™ system (Biotronik™), that enabled daily automatic data transmission, the manual transmissions with the other RPM systems were scheduled in 3 month intervals. The details on the used RPM systems with a brief description of the individual features and the workflow for RPM transmissions can be found in the online supplement. Depending on the assigned RPM system with different initialization requirements patients were instructed over a 30–60-min period about the contents and the usage of the RPM package (monitor, wand, power supply, and telephone cables), including a detailed description of monitor set-up if indicated. In patients with missing transmissions 1 month after enrollment, one single attempt was performed to contact those study participants. All these patients were explicitly notified regarding the failed transmission and with a phone call re-instructed (30 min) on the initializing process.

### Assessment of patient-reported outcomes

Patients were blinded to their assigned study group for baseline assessment of patient-reported outcomes. Patients completed the set of questionnaires on the day after ICD implantation. During the FU period of 12 months, postal surveys were performed on a monthly basis intended to assess discreet changes of patient-reported outcomes and especially to allow for the detection of temporary undulations, e.g., due to cardiac decompensation or ICD shocks [7]. Patients with a missing response (defined as delay > 10 days in expected time of questionnaire return) were contacted by a study nurse (up to three reminder phone calls). After a maximum of three reminder calls without a response, this set of questionnaires was considered lost to follow-up.

### Instruments for assessment of QoL, levels of anxiety/depression, and device acceptance

Outcomes of this trial were measured by the use of patient-reported data obtained from validated questionnaires. To assess QoL, the generic and validated EQ-5D-3L questionnaire by the EuroQol consortium was used [8]. Anxiety and depression levels were measured using the Hospital Anxiety and Depression Scale (HADS-D) [9]. Device acceptance, referring to the psychological accommodation and understanding of the device, was measured using the 18-item Florida Patient Acceptance Survey (FPAS) [10].

#### Health-related quality of life

The generic EuroQol (EQ-5D-3L) questionnaire was designed by the EuroQoL Group as a standardized and generic instrument (cross-disease) for the measurement of health-related QoL. It is often used in combination with disease-specific tools. Generic instruments are designed to measure overall health states and allow comparisons across patients with different diseases and the general population. Therefore, they are suitable to support health policy decisions. The EQ-5D comprises five dimensions (mobility, self-care, usual activities, pain/discomfort, and anxiety/depression) on a three-point scale. The responses indicate three levels of severity (no problems/some or moderate problems/extreme problems) within a particular EQ-5D dimension. Using an algorithm based on eleven European studies, each health state can be transferred into a single index value between 0 (lowest QoL) and 100 (highest QoL) [11]. The ability to convert self-classification responses into a single index score makes the EQ-5D practical for clinical and economic evaluation [12]. Mean levels of QoL in a German cross-section cohort are reported in the literature with 77.4 ± 19 points in the overall population [13] and with 76.29 ± 4.48 points in a subset of elderly people with an age above 65 years, respectively [14]. The average test–retest-reliability is 0.75 (Pearson’s R) [15].

#### Anxiety and depression

Anxiety and depression levels were measured using the Hospital Anxiety and Depression Scale (HADS). The HADS questionnaire consists of 14 items related to anxiety and depression in which patients respond using a 4-point Likert scale [16]. Scores range from 0 to 21 points for both anxiety and depression. With respect to the subscales anxiety and depression, 0–7 indicates non-cases, 8–10 indicates doubtful cases, and 11–21 points indicates manifest depression/anxiety. The reliability/internal consistency (Cronbachs alpha) for the HADS is *α* = 0.8 for both depression and anxiety [17].

#### Device acceptance

Device acceptance refers to the psychological accommodation and understanding of the device and the derivation of benefit in terms of biopsychosocial functioning. Device acceptance was measured using the 18-item Florida Patient Acceptance Survey (FPAS) [10]. The FPAS is one of only a few standardized and validated instruments to measure device acceptance. A 5-point Likert scale was used, ranging from “strongly disagree” (1 point) to “strongly agree” (5 points) [18]. The 18 items contribute to four subscales: (1) return to function, (2) device-related distress, (3) positive appraisal, and (4) body image concerns. The three additional questions are filler items. A higher score indicates greater device acceptance. There are no absolute cut-off values for the FPAS, but based on a prior validation study, a score ≤ 65.5 is considered a correlate of poor device acceptance [18]. The internal consistency ranges from *α* = 0.74 to *α* = 0.89 [10].

### Data analysis and statistical methods

Continuous variables are expressed as means ± standard deviations (SD) unless otherwise noted and categorical variables are presented as frequencies. To exclude sample distortions, all test procedures and sociodemographic variables of the RPM and CTL groups were tested for equality of variances by Levene’s test, and baseline group differences were assessed using independent *t* tests for continuous variables and Chi-square tests for categorical variables.

Unless specified otherwise, all analyses were conducted using univariate ANOVAs with repeated measurement and a Bonferroni post hoc test. To determine the squared sum of the canonical correlations, total Pillai’s trace was calculated. This corresponds to the sum of the “explained” variance in the outcome variable accounted for by the effect of the independent variables. This method was chosen based on its more conservative/robust fashion for smaller sample sizes.

Binary logistic regression models were used to analyze the relative chance of an improvement in QoL between both study arms in pre-defined subgroups to identify patients where additional RPM improved QoL. All changes were measured based on an individual level. Thus, we calculated first the individual mean change over time and then used those individual means to create a group mean.

We calculated odds ratios for the subgroups sex, presence of CRT device, ICD implantation indication (primary vs. secondary prevention), type of enrollment (new implantation vs. generator replacement), and ICD shocks [none vs. any (both appropriate and inappropriate)]. In addition, the median age of 67 years served as cut-off value for the age sub-analysis. A dichotomized QoL variable was created to serve as the outcome variable in the regression models, equaling “QoL increased”, when QoL increased between baseline and FU measurements and “QoL not increased” when mean QoL decreased or remained unchanged. All covariates included in the regression model were selected a priori based on clinical knowledge of their influence on patient-reported outcomes in ICD cohorts. Missing values were processed using a conservative approach and imputed by the last observation carried forward method and last observation carried backward method, respectively. A two-tailed *p*-value of < 0.05 was considered statistically significant.

Patients could only be evaluated if they had at least one questionnaire completed. Our pre-specified modified intention-to-treat (ITT) analysis was, therefore, adjusted accordingly to include all patients who were randomized according to inclusion and exclusion criteria and had completed at least one questionnaire. An additional per-protocol analysis included patients that underwent the intervention (in-office FU with RPM) over the study period of 12 months and performed at least one successful transmission with the RPM system.

The power calculation suggested that for an 80% chance of detecting a 0.05 significant change of quality of life assuming a medium effect size of ƒ = 0.25, 98 subjects would be needed (Fcrit = 3.94). Allowing for a conservative estimate of a 35–40% drop-out (postal survey for a 12 × 1 month period) and a 7% mortality rate, a total sample size of 180 participants had been calculated. Statistical analyses were performed using SPSS for Windows software (Version 24.0; SPSS Institute, Chicago, Ill.) and open source Software R (CRAN-R 3.2.3).

## Results

### Baseline characteristics

The ITT cohort comprised 86 patients in the RPM and 81 patients in the CTL group (see Fig. 1). Table 1 depicts the baseline clinical and demographic characteristics of the study population. Study participants were mainly male (> 80% in both groups); 40.7% and 49.4% had a history of coronary artery disease in the RPM and CTL group, respectively, and the majority of participants (RPM 59.3% vs. CTL 63.0%) were provided with an ICD for primary prevention. About 81% in the RPM and 82.7% in the CTL group were enrolled after de-novo ICD implantation, while the remaining individuals were enrolled for generator replacement. Prior ICD shocks were equally distributed [RPM: mean 0.5; SD: 1.8; CTL: 0.2; SD: 0.8; (*p* = 0.15)]. In the RPM group, only 62 of 86 patients (72%) were able to initialize the system by performing at least one successful transmission.

**Table 1:** Baseline patient characteristics

Baseline	Remote monitoring group		Control group	
Number of subjects (*n*)	86		81	
*Characteristics*				
Male gender, *n* (%)	69	(80.2)	68	(84.0)
Age at implantation, mean (SD)	61.5	(14.2)	63.0	(15.3)
Ejection fraction, mean % (SD)	42.3	(17.0)	37.5	(15.3)
NYHA at implantation, *n* (%)				
1	30	(34.9)	35	(43.2)
2	32	(37.2)	29	(35.8)
3–4	24	(27.9)	16	(19.7)
CRT, *n* (%)	31	(36.0)	29	(35.8)
Primary prevention, *n* (%)	51	(59.3)	51	(63.0)
Type of ICD				
Single and dual chambers, *n* (%)	58	(67.5)	53	(65.5)
CRT defibrillator, *n* (%)	28	(32.6)	28	(34.6)
Device replacements, *n* (%)	16	(18.6)	14	(17.3)
Atrial fibrillation, *n* (%)	32	(37.2)	38	(46.9)
Previous myocardial infarction, *n* (%)	24	(27.9)	23	(28.4)
Stroke, *n* (%)	3	(3.5)	7	(8.6)
Renal insufficiency, *n* (%)	28	(32.6)	34	(42.0)
Chronic obstructive lung disease, *n* (%)	5	(5.8)	6	(7.4)
Diabetes, *n* (%)	16	(18.6)	17	(21.0)
Coronary artery disease, *n* (%)	35	(40.7)	40	(49.4)
Dilated cardiomyopathy, *n* (%)	34	(39.5)	29	(35.8)
Other cardiac condition*, *n* (%)	18	(20.9)	12	(14.8)
Antidepressant medication, *n* (%)	11	(12.8)	9	(11.1)
Smoking behavior				
Active smoker, *n* (%)	9	(10.5)	12	(14.8)
Ex-smoker, *n* (%)	13	(15.1)	23	(28.4)
Patient-reported outcomes				
Quality of Life, mean (SD)	72.9	(21.1)	77.6	(19.9)
Depression, mean (SD)	4.6	(3.9)	3.9	(3.9)
Anxiety, mean (SD)	5.2	(4.0)	4.7	(3.8)
Device acceptance, mean (SD)	72.0	(18.4)	75.4	(16.9)

Values are absolute numbers and percentages unless otherwise indicated

*CRT *cardiac resynchronization therapy, *ICD* implantable cardioverter/defibrillator, *NYHA* denotes New York Heart Association

*Hypertrophic cardiomyopathy; Brugada Syndrome; Long-QT syndrome; Arrhythmogenic right ventricular cardiomyopathy; idiopathic ventricular fibrillation

### Psychological baseline status

Baseline values for mean QoL scores did not differ between the RPM (72.9; SD: 21.1) and CTL group (77.6; SD: 19.9). The mean levels of depression and anxiety were comparable between the RPM and CTL groups (depression: 4.6; SD: 3.9 vs. 3.9; SD: 3.9, *p* = 0.35; anxiety: 5.2; SD: 4.0 vs. 4.7; SD: 3.8, *p* = 0.42, respectively). Baseline values of device acceptance were equally distributed between the RPM and CTL groups (72.0; SD: 16.9 and 75.4; SD: 15.7, *p* = 0.69, respectively, see Table 1).

### Questionnaire return rates

Of the possible 2340 questionnaires that could have been returned (180 patients, 13 each), the return rate was 71%, with 65% of patients having returned more than ten questionnaires. Chi-square tests displayed an equally distributed return rate between the CTL and RPM groups of 70% and 72%, respectively (*χ*^2^ < 1; *p* = n.s.).

### Primary endpoint—changes in QoL over 12 months

**Fig. 2:**
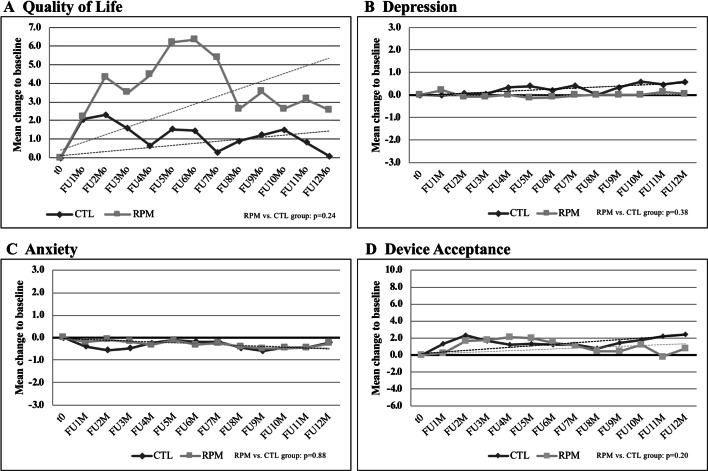
Influence of remote patient monitoring on patient-reported outcomes over 12 months. QoL was not improved by the use of RPM compared to the CTL group (**a**). RPM implementation failed to have significant impact on the levels of depression (**b**), anxiety (**c**), and device acceptance (**d**). *CTL* control, *QoL* quality of life, *RPM* remote patient monitoring;

The analysis of the primary outcome showed a mean change of QoL over 12 months of 1.2 points in the CTL and 3.9 points in the RPM group, respectively. The difference between both groups (intergroup comparison) was not statistically significant (*p* = 0.24, Fig. 2a).

**Fig. 3:**
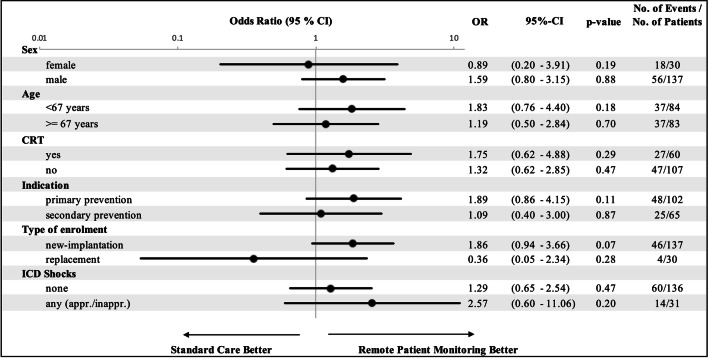
Chance of improvement of QoL depending in clinical/demographic subgroups. No specific subgroup could be identified having significant effect on the impact of additional RPM on QoL. *ICD* implantable cardioverter/defibrillator; *CRT* cardiac resynchronization therapy, *QoL* quality of life

The analysis assessing the impact of the pre-defined subgroups failed to show a significant interaction in one of the subgroups suggesting no difference in outcome by one of the two treatment strategies (Fig. 3). Noteworthy, the sub-analysis regarding type of enrollment showed a trend towards a benefit in patients undergoing de-novo implantation vs. patients after device replacement. A non-pre-specified post hoc sub-analysis (Figure S1, online supplement) assessing the impact of the mode of data transmission revealed that patients equipped with fully automatic transmission RPM systems seem to significantly benefit from this completely user independent, automatic technique (OR 2.52, CI 1.3–5.60; *p* = 0.02), while non-automatic systems with manual transmission are showing a neutral result compared to the CTL arm.

### Changes of QoL within the two study groups (intragroup comparison)

In addition to the analysis of the primary endpoint between both treatment groups (intergroup comparison), we performed a separate intragroup comparison of the QoL change within each group over time vs. baseline. We observed a moderate, but significant increase in QoL within the RPM group (3.9 points, *p* = 0.04), while there was no significant change in QoL in the CTL group (1.2 points, *p* = 0.5) over 12 months (Fig. 2a). This slight increase of 3.9 points in the RPM group could be attributed to the RPM subgroup capable of fully automatic data transmission (Biotronik HomeMonitoring™, mean increase of 7.6 ± 20.0 points).

### Per protocol analysis for the primary endpoint

Based on the fact that 28% of participants never activated RPM, we performed a per-protocol analysis for the patients with a working RPM system. The results of the per-protocol analysis including 62 patients in the RPM group (only patients who had used/activated the RPM) and 81 patients in the CTL group confirmed that an additional FU with successfully initialized RPM did not add any benefit with respect to the primary outcome of QoL in RPM or CTL group (mean change: + 5 points and + 1.2 points, respectively; *p* = 0.63).

### Secondary endpoints

In addition, we aimed to assess the influence of RMP on secondary measures. These analyses revealed no significant group differences for depression, anxiety, and device acceptance (see Fig. 2b–d).

## Discussion

### Relevance of RPM on daily patient care

The INFRARED-ICD trial was specifically designed to investigate the impact of RPM in ICD recipients on patient-reported outcomes. The results of the present study failed to demonstrate a significant benefit of RPM on QoL, levels of anxiety and depression, and device acceptance over the course of 12 months. Furthermore, subgroup analyses failed to identify individuals where the use of RPM improved QoL.

The neutral results of this study with respect to the primary endpoint constitute an important finding since the use of RPM is currently under intense discussion in face of mainly negative results from previous large controlled trials on the effect of RPM on clinical endpoints [5]. The large prospective MORE-CARE study (*n* = 865) failed to show a significant benefit of RPM in CRT patients with respect to mortality and cardiovascular/device-related hospitalizations [4]. The REM-HF study confirmed these results, showing that RPM did not reduce the incidence of cardiovascular events in a heart-failure cohort [5]. In contrast, the IN-TIME and TIM-HF-2 trials were able to show a potential benefit on clinical outcomes by RPM surveillance [19–21]. Regarding economic aspects, RPM bears the potential to be a cost-efficient alternative to regular in-office device interrogations [22, 23]. Given these controversial results, more research on other aspects of RPM is warranted, with high priority on the effect of RPM on factors such as psychological aspects of the patients’ well-being [24–26].

### Baseline measures of patient-reported outcomes

Baselines measures for QoL were 72.9 ± 21.1 and 77.6 ± 19.9 points in the RPM and the control cohort, respectively (Table 1). Comparing those QoL levels with the German overall population (77.4 ± 19 points [13]) suggests no significant impairment of QoL in the study cohort at baseline. In line with these results, baseline measures for anxiety (5.2 ± 4.0 and 4.7 ± 3.8) and depression (4.6 ± 3.9 and 3.9 ± 3.9) in the RPM and the control group did not suggest clinically relevant anxiety/depression levels when compared to the existing literature (German general population: values of 4.4 ± 3.3 and 4.8 ± 4.0 for anxiety and depression, respectively [27]). The levels for device acceptance (72.0 ± 18.4 and 75.4 ± 16.9) for the RPM and control group were also comparable to a reported FPAS score (76.0 points) in a mixed ICD patient cohort [10] and did not suggest any significant impairment of device acceptance at baseline.

### Comparison to previous studies

It is difficult to compare our results to previously published literature, since data on patient-reported outcomes in pre-specified RPM cohorts are scarce [28, 29]. A few randomized trials included QoL as a secondary measure [28, 30–32]. In a sub-analysis of the randomized EVOLVO trial, the authors observed that RPM was associated with a more favorable change in QoL compared to the standard-of-care group [28]. Results from the REFORM trial comparing regular 3-month in-office FUs vs. in-office FU once every 12 months under RPM surveillance support the theory that extended 12-month in-office FUs with additional RPM can result in increased QoL [33]. One single-center pilot study actually demonstrated better outcomes with respect to QoL in patients with more frequent in-office device interrogations vs. sole follow-up by RPM [30]. The authors speculate that the patients’ skepticism about the ease of use of the RPM system and their perception that RPM could impair in-office patient care.

In contrast, a secondary analysis of the MORE-CARE study failed to show a difference in QoL in heart-failure patients who underwent RPM-guided vs. regular in-office FU over a 12-month period [31]. In line with these neutral results, a sub-analysis of the ECOST trial failed to demonstrate a benefit in QoL in the RPM group [32]. The just recently published REMOTE-CIED trial specifically designed to assess health status in a heart-failure cohort confirms the majority of former studies not identifying any specific benefit of RPM surveillance on patient-reported outcomes [6].

Taken divergent results of prior trials, it is important to address the role of different outcome measurement instruments and patient cohorts. The EVOLVO and MORE-CARE trials in heart-failure patients assessed QoL by the Minnesota Living with Heart-Failure Questionnaire, a disease-specific instrument validated in patients with heart failure with preserved and reduced ejection fraction. With respect to RPM studies on mixed patient cohorts, the REFORM [33] and ECOST trials [22] used the SF-36 questionnaire to assess QoL. Although its generic character, the results of the SF-36 cannot be adopted one to one as results of both questionnaires cannot be considered fully interchangeably [34] which further confirms that different outcome measurement instruments and patient cohorts hamper the comparability of the results. It is of crucial importance to keep in mind that the RPM systems used in our study have meanwhile been significantly further developed with respect to ease of use (i.e., initialization process). Especially, the feature of automatic wireless data transmission has meanwhile been applied to each of those systems.

To further understand the discrepancies between previous trials, we analyzed intragroup changes, and observed a significant increase in QoL within the RPM group over the 12-month FU, with just a trend to baseline values in the CTL group. This might suggest a potential benefit with respect to QoL in the RPM group, which could not be confirmed in our intergroup comparison (RPM vs. CTL group) either due to a concomitant QoL improvement of the CTL group or to the low patient number in this study. We were able to show that this slight increase was mainly attributable to the RPM group with automatic data transmission which points at the potential benefit of this novel wireless technique without the need for any user interaction on patient-reported outcomes. This was further supported by our non-pre-specified sub-analysis investigating the impact of the mode of transmission on the individual benefit from RPM. We could show that patients equipped with fully automatic RPM systems seemed to benefit from telemetric data transmission with respect to QoL, while manual transmission showed a neutral result. As meanwhile all ICD manufactures offer wireless RPM solutions capable of fully automatic data transmission, there is the urgent need for future studies aiming to elucidate the effect of RPM systems equipped with the modern-user friendly, automatic features on psychological outcomes.

### Role of patients’ motivation and successful RPM implementation

Studies with negative results on the impact of RPM on clinical endpoints suggest that the lack of patients’ motivation and unsuccessful implementation of the RPM system could be a potential confounder [3, 35]. In the present study, 28% of patients with RPM system failed to initialize the system. Our results are consistent with the previous large randomized trials, showing that 20–24% of patients never activated the RPM system [3, 36]. We, therefore, tested the hypothesis that benefit of RPM on patient-reported outcomes might rely on a working RPM system which could not be confirmed by the results of the per-protocol analysis of our study.

### Secondary endpoints

In addition to the primary outcome, we sought to investigate the effect of RPM on anxiety, depression, and device acceptance. It is hypothesized that the use of RPM has the potential to reduce psychological distress based on studies showing a high patient satisfaction and the feeling of additional safety [23, 29, 37]. Nevertheless, our analysis failed to show a benefit of RPM on the levels of anxiety and depression.

Device acceptance is known to influence the levels of psychological distress as well as QoL, which thus represents a major determinant of psychological well-being in patients with ICDs [18]. In the present study, the intervention of additional RPM in ICD patients did not translate into better device acceptance which suggests that additional technical challenges associated with new technology might lead to mental overload in patients counterbalancing any positive effects.

### Pre-specified subgroup analysis

Since there is evidence that ICD shocks are associated with impaired QoL, we hypothesized that patients with a history of ICD shocks might benefit from RPM [38] which could not be confirmed in the present study. It might be speculated that the overall number of patients with appropriate or inappropriate shocks was too low to provide a significant difference between RPM and CTL group. With respect to implantation indication, our study confirmed results from a previous meta-analysis demonstrating no impact of the initial implantation indication on levels of QoL [39]. Except for pre-existing ICD experience, no significant difference with respect to QoL could be identified in the pre-specified subgroup analyses (patients with vs. w/o ICD shocks; primary vs. secondary implantation indication). In fact, patients undergoing de-novo implantation showed a trend of better QoL by additional RPM. This finding suggests that ICD-naïve patients might be more open-minded towards new techniques potentially simplifying their lives, e.g., by extending the in-office FU intervals or by simply transmitting their data to the hospital in case of suspected events like an ICD shock. Pre-existing ICD FU experience might have blunted the positive effect of RPM as those patients can be assumed to have already adapted well to the implanted device or actually demanded the personal contact to a doctor during an in-office follow-up. This hypothesis has to be further evaluated in future studies.

### Potential relevance and future implications

In addition to available data [3–6], our study adds additional evidence that at the moment it seems hard to justify additional costs for RPM based on the lack of benefit at the moment. However, taking our results on QoL, showing also no negative effect, and taken the beneficial results from the positive In-Time and TIM-HF2 trial, RPM might be at least a very good alternative approach in selected patients. Therefore, the broad implementation of RPM usage might require additional research to identify subgroups of RPM responders or specific diagnostic and therapeutic algorithms related to RPM [19, 20].

### Limitations

Our study has some limitations. As baseline values for QoL assessed by the EQ-5D were unexpectedly high, we cannot rule out the phenomenon of a ceiling effect as the already high baseline value might hamper a significant change in QoL between the groups with the final result of inability to emerge a significant difference between the study groups. The rationale for specifically choosing the EQ-5D was its shortness and simplicity to achieve a high response rate, despite the fact other known disease-specific instruments might have had provided better discrimination abilities for slight differences in QoL.

We only included ICD patients, and therefore, the results may not be applied to pacemaker patients capable of telemetric interrogation. Furthermore, it can be speculated that psychologically distressed patients, specifically those with highest levels of anxiety and depression, declined study participation in terms of a selection bias prior to enrollment.

The short interrogation intervals of 1 month initially intended to assess discreet changes of patient-reported outcomes might constitute a limitation of our study. Such intense interrogations can result in tiring of the participants and making them remember their previous answer which can influence the results.

The rather small patient sample size of this trial and the use of a conservative imputation method (LOCF) as well as the inclusion of patients with only on questionnaire may have contributed to an underestimation of the effect of RPM on patient-reported outcomes, especially in the pre-defined subgroups. Finally, our study results must be seen under the aspect that the current RPM systems have been further developed providing features that were not applicable in all the systems used in our study (i.e., automatic wireless data transmission and ease of use of the initialization process). This is especially important in view of our post hoc finding that the use of the RPM system capable of fully automatic data transmission (Biotronik HomeMonitoring™; *n* = 37 patients) was associated with a significant benefit of RPM usage compared to the CTL group.

## Conclusion

At first glance, results from this randomized study on patient-reported outcomes in ICD recipients suggest no benefit by the use of additional RPM compared to a routine follow-up strategy with respect to QoL, anxiety/depression, and device acceptance. We were unable to identify any specific subgroups of patients who were prone to benefit from RPM implementation. Nevertheless, considering the neutral results of our study, it can be postulated that the approach of RPM-assisted ICD follow-up does not impact QoL in a negative way. Taken that and several other benefits of RPM, which have already been demonstrated (the potential of health care utilization [4] or time to clinical decision reduction [40] or even the chance to improve clinical outcome [19]), this telemetric surveillance could become an alternative approach to standard care with in-office ICD follow-ups. With respect to the design of further studies, it is of crucial importance that they aim at elucidating the benefit of RPM in preselected subgroups as well as at trying to assess the benefit of an exclusive RPM surveillance with no (or at least only annual) in-office device interrogations.

## Electronic supplementary material

Below is the link to the electronic supplementary material.

Supplementary file1 (JPG 741 kb)

Supplementary file2 (DOCX 23 kb)
