# Supplementary figures and images for: The INFluence of Remote monitoring on Anxiety/depRession, quality of lifE, and Device acceptance in ICD patients: a prospective, randomized, controlled, single-center trial
Source: Clin Res Cardiol. 2020 May 16;110(6):789–800. doi: 10.1007/s00392-020-01667-0 (PMC8166667; doi:10.1007/s00392-020-01667-0)

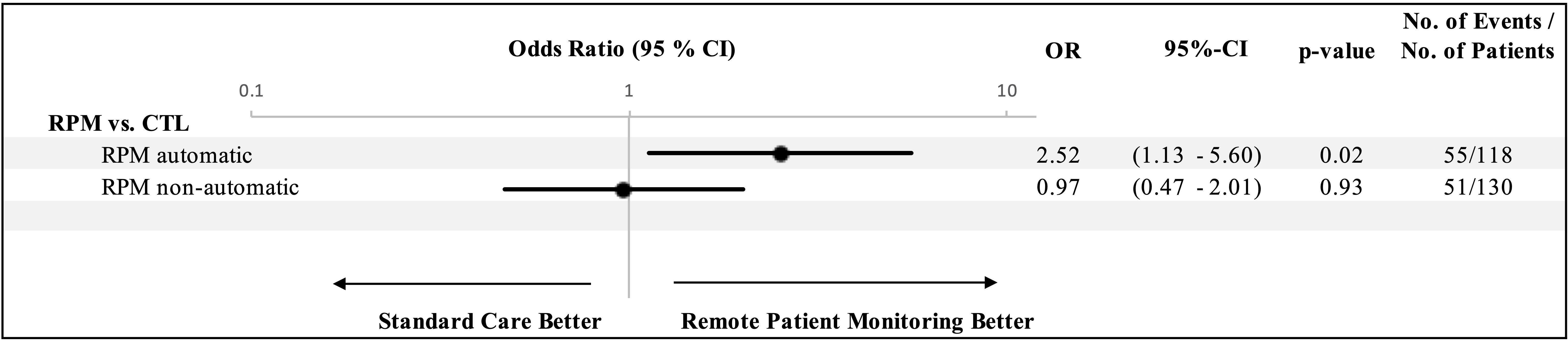

Supplement: Supplementary file 1 — Supplementary file1 (JPG 741 kb) [file 392_2020_1667_MOESM1_ESM.jpg]
