# Supplementary material for: The INFluence of Remote monitoring on Anxiety/depRession, quality of lifE, and Device acceptance in ICD patients: a prospective, randomized, controlled, single-center trial
Source: Clin Res Cardiol. 2020 May 16;110(6):789–800. doi: 10.1007/s00392-020-01667-0 (PMC8166667; doi:10.1007/s00392-020-01667-0)
Supplement: Supplementary file 2 — Supplementary file2 (DOCX 23 kb) [file 392_2020_1667_MOESM2_ESM.docx]

The INFRARED-ICD trial

The INFluence of Remote monitoring on Anxiety/depRession, quality of lifE, and Device acceptance in ICD patients – A prospective, randomized, controlled, single center trial

**Short Title:** QoL in ICD patients with Remote Monitoring

Florian Leppert, MSc.^1^*, Johannes Siebermair, MHBA, MD^2,3,4^*, Ulrich Wesemann, MD^5^, Eimo Martens, MD^2,6^, Stefan M. Sattler, MSc, MD^2,7^, Stefan Scholz, MPH^1^, Stefan Veith^2^, Wolfgang Greiner, PhD^1^, Tienush Rassaf, MD^3^, Stefan Kääb, MD^2,4&^, Reza Wakili, MD^2,3,4&^

1 School of Public Health, Bielefeld University, Bielefeld, Germany

2 Department of Medicine I, University Hospital Munich, Ludwig Maximilians University, Munich, Germany

3 Department of Cardiology and Vascular Medicine, West-German Heart and Vascular Center Essen, University of Essen Medical School, University Duisburg-Essen, Essen, Germany

4 Deutsches Zentrum für Herz-Kreislauferkrankungen (DZHK), partner site Munich Heart Alliance, Munich, Germany

5 Department of Psychiatry, Psychotherapy and Psychotraumatology, Bundeswehr Hospital, Berlin, Germany

6 Medizinische Klinik und Poliklinik, Klinikum rechts der Isar, Technische Universität München, Ismaningerstrasse 22, 81675, Munich, Germany

7 Department of Cardiology, Heart Centre, Copenhagen University Hospital, Rigshospitalet, Copenhagen, Denmark

*contributed equally to the work; ^&^shared senior authorship

**Address for Correspondence:**

Reza Wakili, MD, Department of Cardiology and Vascular Medicine, West-German Heart and Vascular Center, University Duisburg-Essen, Essen, Germany. Tel.: +49-201-723-4803; Fax: +49-201-723-5426; E-mail: reza.wakili@uk-essen.de

**Funding**

This study was supported by public funds of the Bielefeld University, Germany and University Hospital Munich, Ludwig Maximilians University, Munich, Germany.

**Conflict of interest**

All authors declare no conflict of interest.

**Online supplement**

**Supplemental methods and results**

**Brief description of the different systems used in the study:**

- Biotronik

The Home Monitoring^TM^ system (Biotronik, Berlin, Germany) was the first RPM system commercially available, with FDA approval in 2001. The transmission unit (CardioMessenger^TM^) communicated wirelessly with the implanted device. The integrated GSM module enabled complete wireless transmission of the ICD data to a center in Germany without any landline transmission without any user interaction by the treating physician or the patient. After registration on a secured website before discharge the patient just had to connect the CardioMessenger^TM^ at home to the power supply and to turn the transmission unit on [1].

- Medtronic

The usage of the Medtronic RPM system (CareLink^TM^) significantly differed from the Biotronik RPM system during the enrolment period for this study. The main difference to the Biotronik system consisted in the frequency of transmissions, those were not performed automatically on a daily basis but had to be scheduled by the treating physician. Patient equipped with CareLink^TM^ during the study enrolment phase had to manually initialize the system at home, data transmission was performed over a landline requiring active patient involvement. Patients were registered for the RPM system at the discharge visit, with the RPM system postally delivered within 3-7 days including a detailed operating manual. The (first) interrogation and data transmission occurred at the time when the patient placed the interrogation wand over the implanted device [1].

- St. Jude Medical (SJM)

The Merlin.net^TM^ system (St. Jude Medical, now Abbott) has been introduced in 2008. The transmitter (Merlin@Home) communicated wirelessly with the implanted device and sent the interrogation data via an analogue land line to the treating physician. Similar to the CareLink^TM^ system, the further transmission had to be scheduled, automatic daily data transmission was not supported in the version used in this study [1].

- Boston Scientific

Introduced in 2009, the Latitude Patient Management System^TM^ (Boston Scientific, former Guidant) used the analogue land line for data transmission, with wireless transmission of ICD data to the transmitter [1]. After connecting the system to the power supply the participant was led through the installation process to make sure the communicator was installed properly. The other features (online registration, scheduling of transmissions) were similar to the systems provided by Medtronic and SJM [1].

**Workflow for RPM transmissions**

**Description of the device unit**

The transferred data were obtained by the device outpatient clinic of the Department of Cardiology of the LMU Munich. In the regular daily routine two residents were in charge performing the in-office follow-up examinations as well as all follow-up interrogations of the RPM program. All study participants with RPM systems were informed that RPM surveillance did not constitute an emergency system and that they could expect a check of their transmitted data within 3 working days. Therefore, all online RPM data platforms were checked every 1-3 working days. Entries in the specific device calendar assured that scheduled data transmissions could be checked in time.

**Data transmissions**

Regular data transfers were either scheduled transmissions (CareLink^TM^; Latitude^TM^; and Merlin.net^TM^ systems) or daily automatic transmissions (HomeMonitoring^TM^). In addition, the study participant could trigger patient-initiated (manual) transmission in case of any unexpected event (shock delivery, sound notification of the device). For those cases of unexpected events with manual data transmission patients were provided with a direct phone number of our device outpatient clinic.

**Processing of incoming data and feedback to the patients**

There was a standard on how to proceed with incoming data transmissions. Incoming transmissions were checked if they comprised regular (scheduled) or patient-triggered transmission in case of suspected events:

- Regular transmission:

a) Proper device function: A short medical report was sent to the patient and his family doctor

b) Device malfunction/battery depletion: A rating of the severity of the event was performed to decide whether a direct admission of the patient to the hospital with immediate feedback by a phone call was indicated

c) Recorded arrhythmia events: The decision on how to proceed was based on the severity of the event (supraventricular vs. ventricular arrhythmia, sustained vs. non-sustained, heart rate etc.). In case of life-threatening arrhythmias, the patients were immediately contacted

- Patient-triggered transmission:

Arrhythmia events and device (mal)function: The patients were contacted by phone or mail and informed about the status of the device function or subjective arrhythmia episodes

**The impact of the mode of data transmission on the endpoint “QoL improved”**

The intragroup analysis (Table S1) with the respect to change of QoL over time showed that the significant increase within the RPM group was mostly attributable to patients with fully automatic RPM systems (mean increase of 7.6 ± 20.0 points, p=0.03) when compared to the manual transmission RPM group (1.0 ± 14.2, p=0.6). Based on this finding we conducted a non-prespecified post-hoc subanalysis assessing the impact of the mode of data transmission on the endpoint “QoL increased”. This subanalysis (Figure S1) could demonstrate a significant interaction revealing that automatic transmission was associated with better QoL with respect to the dichotomized endpoint (OR 2.52; CI 1.13-5.60, p=0.02).

**Table S1**

**Results for Quality of Life:**

|  | p-value of  **intragroup change** | | p-value of  **intergroup change** |
| --- | --- | --- | --- |
| **Biotronik RPM patients  vs. CTL group** | Biotronik RPM (n=37) | CTL group (n=81) |  |
| **Mean change over 12 months** | 7.6 ± 20.0 (p=0.03) | 1.2 ± 16.4 (p=0.51) | p=0.10 |
|  | | | |
|  | p-value of  **intragroup change** | | p-value of  **intergroup change** |
| **Biotronik RPM patients  vs. all other RPM patients** | Biotronik RPM (n=37) | Other RPM (n=49) |  |
| **Mean change over 12 months** | 7.6 ± 20.0 (p=0.03) | 1.0 ± 14.2 (p=0.6) | p=0.26 |

**References**

1. Burri H, Senouf D (2009) Remote monitoring and follow-up of pacemakers and implantable cardioverter defibrillators. Europace 11 (6):701-709. doi:10.1093/europace/eup110

**Figure legend**

**Figure S1. The impact of the mode of data transmission on the endpoint “QoL improved”** This non-prespecified subanalysis showed that patients equipped with a RPM system capable of fully automatic data transmission without any user interaction significantly benefitted from the RPM transmission with respect to QoL (OR 2.52, p=0.02)
